# Two novel *KCNA1* variants identified in two unrelated Chinese families affected by episodic ataxia type 1 and neurodevelopmental disorders

**DOI:** 10.1002/mgg3.1434

**Published:** 2020-07-23

**Authors:** Haiming Yuan, Huihua Yuan, Qingming Wang, Wanhua Ye, Ruixia Yao, Wanfang Xu, Yanhui Liu

**Affiliations:** ^1^ Dongguan Maternal and Child Health Care Hospital Dongguan China; ^2^ Dongguan Institute of Reproductive and Genetic Research Dongguan China

**Keywords:** episodic ataxia type 1, *KCNA1*, low‐level mosaicism, neurodevelopmental disorder, seizures

## Abstract

**Background:**

Pathogenic *KCNA1* variants have been linked to episodic ataxia type 1 (EA1), a rare neurological syndrome characterized by continuous myokymia and attacks of generalized ataxia that can be triggered by fever, abrupt movements, emotional stress, and fatigue. Currently, over 40 *KCNA1* variants have been identified in individuals with EA1.

**Methods:**

A male patient displayed partial seizures in addition to EA1 symptoms, often triggered by fever. A sibling presented with typical EA1 symptoms, seizures, and learning difficulties. In addition, the older brother displayed cognitive impairment, developmental delay, and slurred speech, which were absent in his younger sister. Whole‐exome sequencing was performed for the patients.

**Results:**

A novel *de novo* missense variant in *KCNA1* (p.Ala261Thr) was identified in the male patient, which is located in a base of the 3rd transmembrane domain (S3). The other novel *KCNA1* variant (p.Gly376Ser) was identified in the sibling and was inherited from an unaffected father with low‐level mosaicism. The variant was located in the S5–S6 extracellular linker of the voltage sensor domain of the Kv channel. Next, we systematically reviewed the available clinical phenotypes of individuals with EA1 and observed that individuals with *KCNA1* variants at the C‐terminus were more likely to suffer from seizures and neurodevelopmental disorders than those with variants at the N‐terminus.

**Conclusion:**

Our study expands the mutation spectrum of *KCNA1* and improves our understanding of the genotype–phenotype correlations of *KCNA1*. Definitive genetic diagnosis is beneficial for the genetic counseling and clinical management of individuals with EA1.

## Graphical abstract

Mutations in the KCNA1 gene encoding the Kv1.1 voltage‐gated potassium (K+) channel have been linked to episodic ataxia type 1 (EA1), a rare neurological syndrome characterized by continuous myokymia and attacks of generalized ataxia that can be triggered by fever, abrupt movements, emotional stress, and fatigue. Here, two novel KCNA1 variants (p.Ala261Thr and p.Gly376Ser) were identified in one male patient and a sibling from two unrelated Chinese families. Next, we systematically reviewed the available clinical phenotypes of individuals with EA1 and observed that individuals with KCNA1 variants at the C‐terminus were more likely to suffer from seizures and neurodevelopmental disorders than those with variants at the N‐terminus. Thus, our study expands the mutation spectrum of KCNA1 and improves our understanding of the genotype‐phenotype correlations of KCNA1. Definitive genetic diagnosis is beneficial for the genetic counseling and clinical management of individuals with EA1.

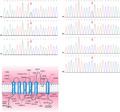

## 1 INTRODUCTION

Episodic ataxia type 1 (EA1) is an autosomal dominant potassium channelopathy characterized by constant myokymia and paroxysmal ataxia that may be triggered by sudden movement, fever, startle, stress, and fatigue (VanDyke, Griggs, Murphy, & Goldstein, 1975). EA1 patients have been reported with unusual phenotypes such as episodic ataxia without myokymia, distal lower limb weakness and stiffness, joint contractures, skeletal deformities, paroxysmal dyspnea (Butler, da Silva, Alexander, Hegde, & Escayg, 2017; Imbrici et al., 2017; Klein, Boltshauser, Jen, & Baloh, 2004; Lee et al., 2004; Shook, Mamsa, Jen, Baloh, & Zhou, 2008), and neurodevelopmental anomalies including epilepsy, cognitive impairments, and developmental delay (Butler et al., 2017; Demos et al., 2009; Eunson et al., 2000; Rogers et al., 2018; Tristán‐Clavijo et al., 2016; Trujillano et al., 2017; Yin et al., 2018; Zhu, Alsaber, Zhao, Ribeiro‐Hurley, & Thornhill, 2012; Zuberi et al., 1999).

EA1 is caused by the loss‐of‐function (Rea, Spauschus, Eunson, Hanna, & Kullmann, 2002; Scheffer et al., 1998) or dominant‐negative mutations (Eunson et al., 2000; Rogers et al., 2018; Tristán‐Clavijo et al., 2016; Yin et al., 2018; Zuberi et al., 1999) of the *KCNA1* gene (MIM 176260), encoding the Kv1.1 voltage‐gated delayed potassium (K+) channel, that lead to neuronal hyperexcitability. This delayed rectifier K+ channel is composed of four homologous alpha subunits, each comprising six transmembrane segments (S1–S6), and can be assembled as a homomeric or heteromeric protein structure with other members of the same subfamily. The S5–S6 segments and the loop between S5 and S6 are a part of the ion‐conducting pore of the channel and provide the selectivity filter for K+. The S1–S4 segments form the voltage‐sensor domain, which is coupled with the helical S4–S5 linker to the potassium pore (Kuang, Purhonen, & Hebert, 2015). Clinical heterogeneity has been reported among patients with the same *KCNA1* mutation and has even been observed between identical twins (D'Adamo et al., 2015). To date, more than 40 mutations in *KCNA1* have been identified in patients with EA1 (Hasan & D'Adamo, 2018; Imbrici et al., 2008; Rogers et al., 2018; Tomlinson et al., 2010; HGMD). Here, we identified two novel *KCNA1* variants (p. Ala261Thr and p. Gly376Ser) in the two unrelated Chinese families affected by EA1 and neurodevelopmental disorders.

## 2 MATERIALS AND METHODS

### 2.1 Ethical compliance

This study was approved by the Ethics Committee of Dongguan Maternal and Child Health Care Hospital. Written informed consent was obtained from the families.

### 2.2 Whole‐exome sequencing

Whole‐exome sequencing (WES) of the patients was performed to screen for causal variants. Sequencing was performed on the NextSeq500 platform (Illumina) according to the manufacturer's protocols. Clinic Sequence Analyzer (CSA) software was used for biological analysis and interpretation. The pathogenicity of the sequence variants was interpreted in accordance with the American College of Medical Genetics and Genomics/Association for Molecular Pathology (ACMG/AMP) guidelines (Richards et al., 2015).

## 3 RESULTS

### 3.1 Patient 1

The male patient is the only child of healthy, nonconsanguineous Chinese parents, and was born at 38 weeks gestation following an uneventful pregnancy. He exhibited normal birth measurements: weight 3.2 kg (38.2%), length 50 cm (52.4%), and head circumference 34.5 cm (51.2%). His Apgar scores were all 10. At 1 year and 9 months of age, he first presented with partial seizures. The events lasted for 40 seconds each time, with spontaneous remission. The patient was conscious throughout the seizures. The seizures were triggered by fever, fatigue, and illness. Seizure occurrence was often accompanied by episodic ataxia and myokymia. The patient was treated with carbamazepine and showed no further epileptic seizures.

At the age of 2 years and 8 months, seizures were again induced by fever, which was characterized by upturned eyes, closed teeth, clenched fists, flexed and twitching limbs, and myokymia. Consciousness was preserved during the attack. The seizures lasted for 1–2 minutes each time with spontaneous remission. Routine electroencephalography (EEG) showed extensive mild abnormalities without obvious epileptic discharge. The patient was treated with carbamazepine and exhibited an excellent response, and it was reported that his seizures completely disappeared within one month of starting the medication. Then, carbamazepine was discontinued, and he presented no further episodes until the age of 3 years and 5 months when he exhibited a brief staring spell followed by flaccidity of the arms and legs. The event was also triggered by fever.

Upon the last physical examination at the age of 3 years and 10 months, the patient's growth parameters were normal, and no dysmorphic facial features were recognized. He showed normal cognitive ability. His developmental milestones were within the normal range: he raised his head at 2 months, sat alone at 6 months, and walked independently at 1 year and 2 months of age. He exhibited normal language development. Brain magnetic resonance imaging (MRI) was normal.

**Figure 1:**
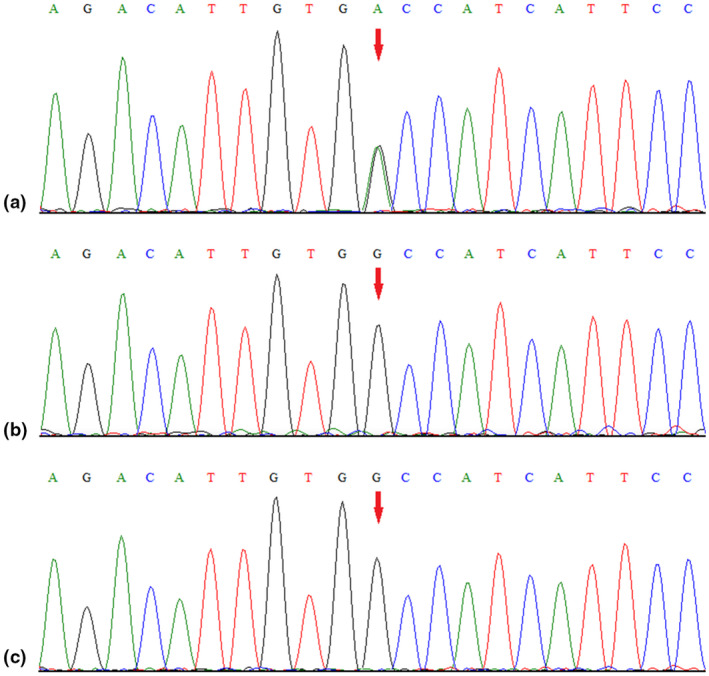
Sanger sequencing results for the patient (a), and the patient's father (b), and mother (c). The analysis demonstrated the presence of a missense *KCNA1* variant (c.781G>A, p.Ala261Thr; red arrow) in the patient and the absence of the variant in her parents

A novel heterozygous missense *KCNA1* variant (NM_000217.2:c.781G>A, p.Ala261Thr) was identified in this patient. Subsequent targeted Sanger sequencing confirmed the *de novo* origin of the variant (Figure 1). This variant occurs in the highly conserved pore region of the Kv1.1 voltage‐gated potassium channel. It was not present in either the Genome Aggregation Database or 1000 Genomes Project database and was predicted to have a deleterious effect on the gene product by multiple *in silico* prediction tools [MutationTaster, SIFT and PolyPhen‐2]. Thus, this variant was categorized as clinically likely pathogenic according to ACMG/AMP guidelines (PS2+PM1+PM2+PP3) (PS: pathogenic strong; PM: pathogenic moderate; PP: pathogenic supporting).

### 3.2 Patients 2 and 3

A sibling was born at full term via spontaneous vaginal delivery after an uneventful pregnancy with weight, height, and head circumference well within the normal ranges. There was no family history of seizures or other genetic disorders. At 11 months of age, the older brother was diagnosed with seizures, which responded well to Depakine. His growth development was normal, but his motor development was delayed: he walked without assistance at the age of 1.8 years. He displayed slightly delayed language development, dysarthria and cognitive impairment, and learning difficulties with Intelligence Quotient (IQ) 75 by the Wechsler Intelligence Scale. He had recurrent headaches and dizziness. At 24 years of age, his seizures were under good control with Depakine, although he did still have occasional tonic‐clonic seizures, attacks of generalized ataxia, neuromyotonia, myokymia, tremor, dizziness or diplopia. These events were often triggered by fever, abrupt movement, physical exercise, fatigue, emotional stress or even sudden changes in temperature. He showed no distinctive facial features but appeared dull and clumsy. Brain MRI results were normal.

The younger sister was born with normal birth measurements: weight 3.3 kg (56.0%), length 49 cm (46.8%), and head circumference 34.0 cm (54.0%). She exhibited normal developmental milestones. She was referred to the clinic because of seizures at 3 years of age. Her seizures were characterized by absence spells and lasted for approximately 30 seconds each time, with spontaneous remission, and were usually accompanied by continuous myokymia and short attacks of episodic ataxia. The events were often triggered by fever, physical exercise, fatigue or emotional stress. As of the age of 21 years, she had reached a nearly seizure‐free state, with Depakine being the most effective drug, and exhibited occasional seizures, which were often triggered by the inducing factors mentioned above. Her cognitive competence was not impaired, but she exhibited learning difficulties. She showed normal language development and did not display slurred speech, as did her older brother. No abnormalities were observed in her brain MRI.

**Figure 2:**
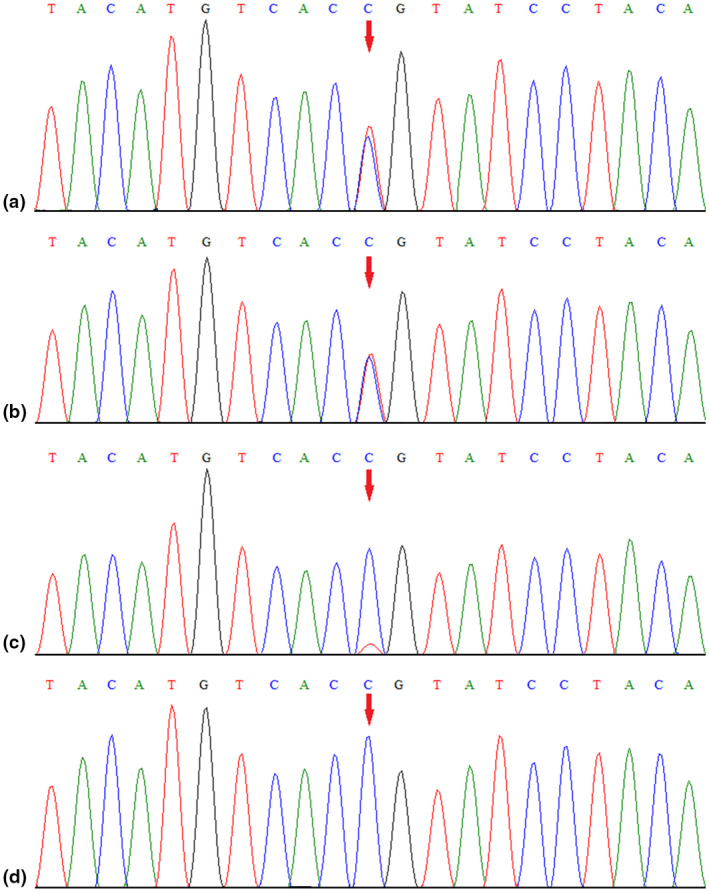
Sanger sequencing results for the younger sister (a), the older brother (b) and the sibling's father (c), and mother (d). The analysis revealed the presence of a missense *KCNA1* variant (c.1126G>A, p. Gly376Ser; red arrow) in the sibling and the absence of the variant in the mother, and confirmed that the variant was inherited from the unaffected father with low‐level mosaicism

WES identified a novel *KCNA1* variant (NM_000217.2: c.1126G>A, p.Gly376Ser) in the sibling. This variant was proven to be inherited from the unaffected father, who exhibited low‐level mosaicism, by parental targeted Sanger sequencing (Figure 2). This variant occurred in the highly conserved S5–S6 extracellular linker of the Kv1.1 voltage‐gated potassium channel. This variant was not present in either the Genome Aggregation Database or 1000 Genomes Project and was predicted to be damaging by multiple *in silico* tools [MutationTaster, SIFT and PolyPhen‐2]. Thus, this variant was clinically categorized as likely pathogenic according to ACMG/AMP guidelines (PS2+PM1+PM2+PP3) (PS: pathogenic strong; PM: pathogenic moderate; PP: pathogenic supporting).

## 4 DISCUSSION

Pathogenic *KCNA1* variants have been reported in patients with EA1 characterized by constant myokymia, episodic ataxia, and occasional epilepsy but normal cognition, and were unexpected in patients with severe epilepsy and significant cognitive impairment (Rogers et al., 2018). In this study, the male patient was first brought to our clinic at the age of 1 year 9 months because of exhibiting partial seizures in addition to EA1. The events were mainly induced by fever and showed an excellent response to carbamazepine. A novel *de novo* variant in *KCNA1* (p. Ala261Thr), positioned in the 3rd transmembrane domain (S3), was identified in our patient. He showed normal cognitive competence and developmental milestones, although he still occasionally suffered from seizures at the time of this report. To date, four pathogenic *KCNA1* variants (Asn255Lys, Asn255Asp, Ile262Thr, and Ile262Met) have been identified in the 3rd transmembrane domain (S3). The patients carrying these variants have only presented with EA1 symptoms. None of these patients was clinically diagnosed with seizures (Klein et al., 2004; Lassche et al., 2014; van der Wijst, 2018; Yin et al., 2018). In this study, the novel nearby *KCNA1* variant (p.Ala261Thr) identified in our patient is the first reported to be associated with seizure located in the 3rd transmembrane domain (S3).

The sibling was referred for genetic counseling clinic because they exhibited strikingly similar clinical phenotypes. Both parents were apparently healthy and the family history was unremarkable. The subjects had reached the age of marriage and were eager to understand the causes and reproductive risks that they faced. WES was performed for the sibling and identified a novel missense variant (p.Gly376Ser), positioned in the S5–S6 extracellular linker of the voltage sensor domain of the Kv channel (Kuang et al., 2015). This variant was confirmed to be inherited from an unaffected parent with low‐level mosaicism and was classified as clinically likely pathogenic according to ACMG/AMP guidelines. Currently, few pathogenic variants in the S5–S6 linker have been reported in individuals with EA1. Thus, little is known about the clinical consequences of variants in this domain. Recently, Verdura et al. (2020) identified a homozygous pathogenic *KCNA1* variant (p.Val368Leu), positioned in the S5–S6 linker, in a patient presenting with seizures and neurodevelopmental disorders in addition to EA1 symptoms, which was the first to be reported to act in a recessive mode of inheritance in *KCNA1*. The sibling described herein experienced typical EA1 symptoms including episodic ataxia and constant myokymia and unusual phenotypes such as seizures and learning difficulties. It has been previously reported that the severity of clinical presentation may differ among patients carrying *KCNA1* variants, even between patients with the same mutation (D'Adamo et al., 2015). In this study, the sibling carried the same mutation but exhibited clinical phenotypes with varying degrees of severity. The older brother displayed cognitive impairment and delayed motor and language development, whereas his younger sister did not show these features but exhibited learning difficulties. Both siblings exhibited seizures, but the age of onset for the older brother was earlier than that for the younger sister, and the older brother was more severely affected than the younger sister. Both responded well to Depakine without side effects, although they still experienced occasional seizures, often triggered by fever, physical exercise, fatigue, emotional stress or illness. In general, both the severity and prognosis of the younger sister's clinical manifestations were more favorable than those of the older brother.

**Figure 3:**
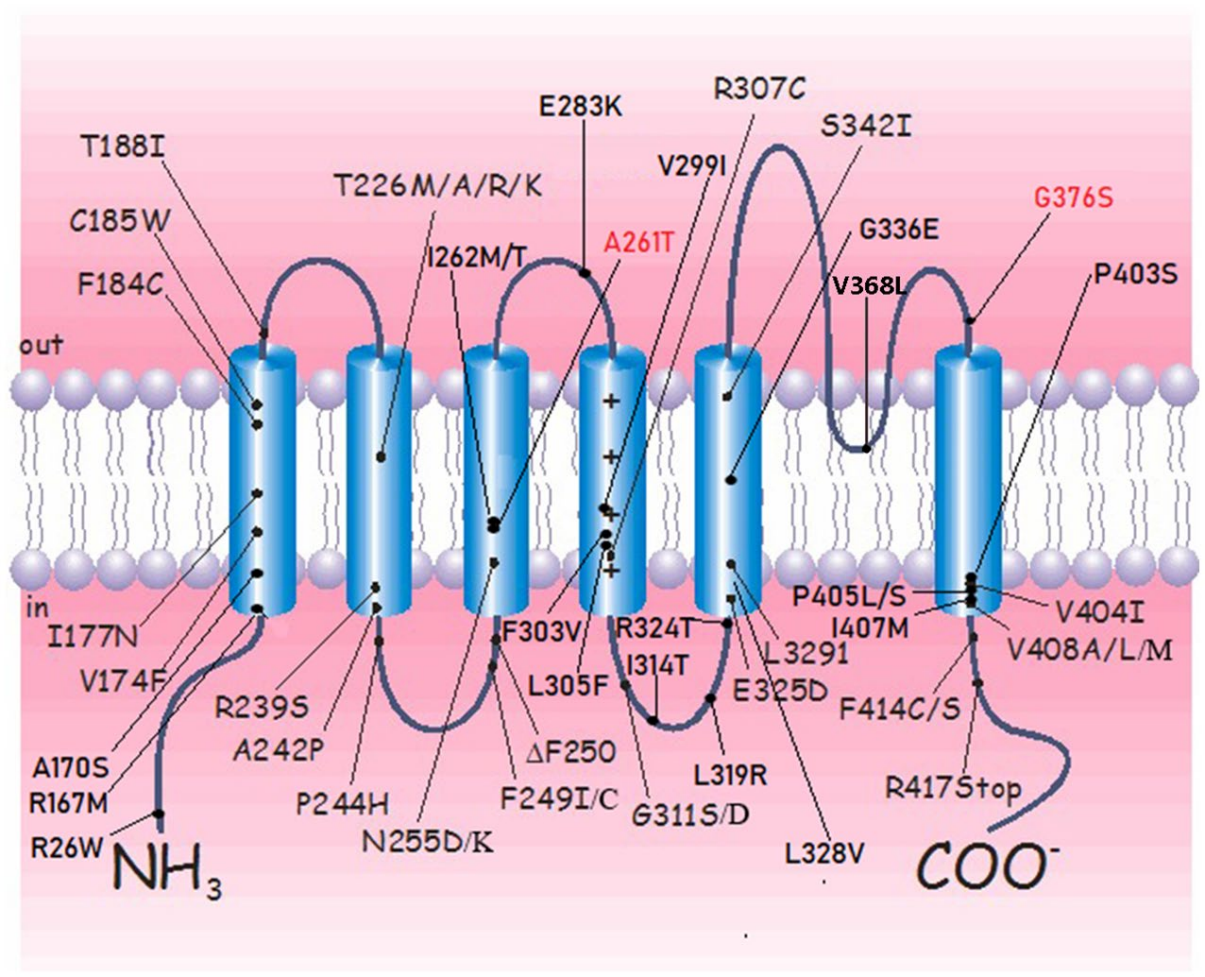
Schematic representation of *KCNA1* variants identified to date in individuals with EA1, which was adapted from Hasan et al. This delayed rectifier K+ channel included four homologous alpha subunits. Each subunit comprises six transmembrane segments (S1–S6), and can be assembled as a homomeric or heteromeric protein structure with other members of the same subfamily. The S5–S6 segments and the loop between S5 and S6 are part of the ion‐conducting pore of the channel and provide the selectivity filter for K+. The S1–S4 segments form the voltage‐sensor domain, which is coupled with the helical S4–S5 linker to the potassium pore. Black: Variants reported in the literature; Red: Variants reported in this study

Next, we systematically reviewed all *KCNA1* variants and related phenotypes from the medical literature. The distribution of variants in *KCNA1* and functional regions of the protein are depicted in Figure 3. It has been reported that *de novo KCNA1* variants (Pro403Ser; Val404Ile; Pro405Leu) in the PVP motif in the 6th transmembrane domain (S6) abolish channel function and cause infantile epileptic encephalopathy and cognitive impairment similar to recurrent *KCNA2* variants (Rogers et al., 2018). At least two nearby *KCNA1* variants (p.Val408Met and p.Val408Leu) have also been associated with variable cognitive impairment and epilepsy (Butler et al., 2017; Demos et al., 2009). A novel homozygous *KCNA1* variant (p.Val368Leu) in the S5–S6 linker has recently been reported in a patient with neurodevelopmental disorders (Verdura et al., 2020). The sibling described in the present study carrying the variant (p.Gly376Ser) in the same domain presented with variable cognitive impairment and epilepsy as well. The variants described above are concentrated in the C‐terminal domain of the KCNA1 protein and contribute to neurodevelopmental anomalies. Individuals carrying the Leu319Arg, Arg324Thr, Gly336Glu, and Ser342Ile variants have been reported to exhibit epilepsy in addition to EA1 symptoms but show no cognitive impairment or developmental delay (Tristán‐Clavijo et al., 2016; Trujillano et al., 2017; Yin et al., 2018; Zhu et al., 2012). These variants are located in the 5th transmembrane domain (S5) except for the variant (Leu319Arg) in the S4–S5 linker. However, individuals with variants located in N‐terminal domains usually show EA1 symptoms and only sporadic seizures, associated with two variants (p.Thr226Arg, p.Ala242Pro) positioned in the 2nd transmembrane domain (S2) (Eunson et al., 2000; Zuberi et al., 1999). Previously reported individuals with EA1 and epilepsy have shown to harbor dominant‐negative *KCNA1* variants, suggesting that epilepsy might be related to a more severe disturbance of K1 channel function (Lassche et al., 2014). Most of the variants discussed above have been experimentally confirmed to cause disease through a dominant‐negative mechanism, which further supports the previous hypothesis. Furthermore, we observed that the phenotypes resulting from variants at the C‐terminus are more likely to be related to neurodevelopmental disorders and are more severe than those resulting from variants at the N‐terminus. This suggests that variants in the S5–S6 segments and the linker between S5 and S6, playing a role in the ion‐conducting pore of the channel and providing the selectivity filter for K+, may exacerbate the phenotype. The identification of the genotype–phenotype relationship would aid in the prognostic evaluation and clinical management of individuals with EA1.

In conclusion, we identified two novel *KCNA1* variants (p.Ala261Thr and p.Gly376Ser) in two unrelated Chinese families presenting variable neurodevelopmental anomalies in addition to EA1 symptoms. Our study expands the known mutational spectrum of the *KCNA1* gene. By comparing the genotype–phenotype relationships of all patients with *KCNA1* variants, it was observed that the phenotypes resulting from variants at the C‐terminus are more likely to be related to neurodevelopmental disorders. These findings will be valuable in the genetic counseling and clinical management of patients with EA1.

## CONFLICT OF INTEREST

The authors declare no conflict of interest.

## AUTHORS’ CONTRIBUTIONS

Haiming Yuan was responsible for the design of the project and drafted the versions of the manuscript. Wanfang Xu and Yanhui Liu were responsible for data analysis, clinical assessment, and the design of the project. Huihua Yuan, Qingming Wang, Wanhua Ye, and Ruixia Yao assisted in the clinical assessment and participated in the experiments, data entry, and data analysis phases of the project. All authors have read and approved the final version of the manuscript.
